# Psychoeducation for adults with type 1 diabetes and problematic hypoglycemia: implementation analysis of a clinical trial (HARPdoc)

**DOI:** 10.3389/frhs.2026.1792881

**Published:** 2026-05-19

**Authors:** Tayana Soukup, Samantha Cross, Stephanie A. Amiel, Kimberley Goldsmith, Augustin Brooks, Linda Gonder Frederick, Andy Healey, Simon Heller, Louise Hull, Dulmini Kariyawasam, Sabine Landau, Laura Potts, Helen Rogers, Emma L. Smith, Elena Toschi, Nicole de Zoysa, Lis Warren, Nick Sevdalis, Ioannis Bakolis

**Affiliations:** 1 Centre for Mental Health Policy and Evaluation, Health Service and Population Research Department, Institute of Psychiatry, Psychology and Neuroscience, King's College London, London, United Kingdom; 2 Department of Surgery and Cancer, Faculty of Medicine, Imperial College London, London, United Kingdom; 3 Department of Biostatistics & Health Informatics, Institute of Psychiatry, Psychology and Neuroscience, King’s College London, London, United Kingdom; 4 Department of Diabetes, School of Cardiovascular and Metabolic Medicine and Sciences, King’s College London, London, United Kingdom; 5 Department of Diabetes, King’s College Hospital NHS Foundation Trust, London, United Kingdom; 6 University Hospitals Dorset NHS Foundation Trust, Bournemouth, United Kingdom; 7 Department of Psychiatry and Neurobehavioral Sciences, University of Virginia, Charlottesville, VA, United States; 8 Division of Clinical Medicine, School of Medicine and Public Health, University of Sheffield, Sheffield, United Kingdom; 9 Diabetes Research Centre, College of Life Sciences, University of Leicester, Leicester, United Kingdom; 10 Department of Diabetes and Endocrinology, Guy’s and St Thomas’ NHS Foundation Trust, London, United Kingdom; 11 Joslin Diabetes Center, Harvard Medical School, Boston, MA, United States; 12 On Behalf of the HARPdoc Trial Patient Advisory Group, Department of Diabetes, King’s College London, London, United Kingdom; 13 Center for Behavioral and Implementation Science Interventions, Yong Loo Lin School of Medicine, National University of Singapore, Clementi, Singapore

**Keywords:** acceptability, appropriateness, feasibility, hypoglycemia awareness, implementation science, patient education programs, type 1 diabetes

## Abstract

**Background:**

The Hypoglycemia Awareness Restoration Program for people with type 1 diabetes and problematic hypoglycemia with severe episodes persisting despite optimal care (HARPdoc) uniquely focusses on addressing cognitive and motivational barriers to hypoglycemia avoidance associated with impaired awareness to hypoglycemia. We aimed to compare perceptions of acceptability, feasibility, and appropriateness of HARPdoc intervention to an existing program, Blood Glucose Awareness Training (BGAT) and understand how these implementation outcomes relate to cognitive and mental health clinical outcomes.

**Methods:**

The HARPdoc trial was a hybrid randomized clinical trial delivered in the United Kingdom and United States between July 2018 and December 2019. Implementation outcomes, including perceived acceptability, appropriateness, and feasibility, were measured using published validated surveys. These surveys were completed by the people with diabetes, healthcare professionals, and relatives of participants. Clinical outcomes, including attitudes to awareness, diabetes distress, anxiety, and depression, were measured using validated self-reported questionnaires. We explored differences of perceived implementation outcomes between HARPdoc and BGAT and associations between implementation and clinical outcomes using quantile and linear regression. We also assessed whether the effect of HARPdoc on cognitive and mental health outcomes were mediated by implementation outcomes.

**Results:**

HARPdoc was perceived as more appropriate than BGAT at 12 months, with a median difference of 0.75 (95% CI 0,26,1,24) by both those involved in delivering the programs and the HARPdoc participants. All stakeholder groups also perceived HARPdoc intervention as more acceptable (MD 0.50 95% CI 0.13, 0.87), but as feasible (MD 0.00; 95% CI −0.31, 0.31) as BGAT. Each of perceived acceptability, appropriateness, and feasibility were significantly linked to improvements in clinical outcomes (feasibility- anxiety: Mean Difference: −1.07; 95% CI: −2.03, −0.10); feasibility-depression (MD: −5.25, 95% CI −9.09, −1.41)). No evidence of mediation was observed.

**Conclusions:**

HARPdoc compared to BGAT was perceived as more appropriate and acceptable and subsequently higher perceived appropriateness, acceptability and feasibility was linked to better cognitive and mental health outcomes. Our findings provide important insights for the development of an implementation blueprint and the expansion of HARPdoc and BGAT programs into routine healthcare services and highlight the need for larger, better-powered hybrid trials.

## Introduction

Type 1 diabetes (T1D) is a condition characterized by deficient insulin secretion, resulting in high blood glucose levels and always requiring exogenous insulin treatment (1). Insulin therapy for T1D carries the risk of severe hypoglycemia (SH), where blood glucose drops dangerously low, causing cognitive impairment (2, 3). Each year, 22%–46% of individuals with T1D experience SH, leading to potential mortality and significant healthcare burden (4) Impaired awareness of hypoglycemia (IAH) is a significant factor in managing hypoglycemia in adults with T1D. It refers to reduced intensity or delayed onset of symptoms that prompt glucose intake and is associated with a greatly increased risk of SH. Studies indicate that 25%–40% of adults with T1D experience IAH (3), even among those using state-of-the-art technology for glucose monitoring (5) and insulin delivery (6).

Structured education programs like Diabetes treatment and teaching programs (DTTPs) (7), Beta Cell Education Resources for Training in Insulin and Eating (BERTIE) (8), and Dose Adjustment For Normal Eating (DAFNE) (9) aim to provide knowledge and skills in insulin management to adults with T1D. Blood Glucose Awareness Training (BGAT) is a psycho-educational program which additionally provides skills in predicting and preventing high and low blood glucose values (10). These programs offer general training in diabetes self-management, including hypoglycemia prevention and, while not explicitly focusing on IAH or SH, do reduce hypoglycemia risk (11–14). However, not all participants in such programs regain hypoglycemia awareness and residual SH is reported (10, 14). IAH and recurrent SH are reported in 30% and 9% of people using automated insulin delivery systems (hybrid closed loop) respectively (6). Targeted approaches may be necessary for individuals experiencing persistent problematic hypoglycemia despite education with or without technology. One such program, named HyPOS, a structured educational intervention specifically developed to address hypoglycaemia problems and improve awareness and self-management in individuals with type 1 diabetes, significantly reduced severe hypoglycemia compared with a general education control program (15). However, the impact of a hypoglycemia-targeted program following an unsuccessful attempt with a more generic education program has not yet been explored.

More recently, a novel program has been developed to help restore hypoglycemia awareness in people who have been through structured education and have had access to technology. Following a successful pilot (DAFNE-HART), the program was finalized as HARPdoc (Hypoglycemia Awareness Restoration Program for adults with type 1 diabetes and hypoglycemia persisting despite optimized care) (16–18). A unique feature of the HARPdoc intervention is its focus on addressing the cognitive and motivational barriers associated with impaired awareness through the utilization of established psychological techniques (16–18). It has been compared to BGAT in a randomized clinical trial where both had similar effects in reducing SH and improving SH awareness (19). There were however differences between the programs in secondary outcomes relating to psychological wellbeing, including scores for diabetes distress, general anxiety, and depression (19), all since found to be higher in groups with IAH (20, 21). HARPdoc intervention has also been found cost-effective compared to BGAT through outcomes not directly related to hypoglycemia (22).

To facilitate timely and effective integration of the findings into practice, the HARPdoc trial was designed as hybrid study with equal emphasis placed on investigating the clinical effectiveness and implementation aspects of HARPdoc intervention compared to BGAT (16–18). Such studies have the potential to expedite the implementation process by building a comprehensive knowledge base around clinical processes, systems, implementation determinants and implementation strategies, ultimately supporting the adoption, implementation, and long-term utilisation of innovative practices in real-world healthcare settings, following positive trial results (23–28). We followed the guidance of the Medical Research Council (24–27) and Damschroder and Hagedorn (29) in exploring the relationship between program implementation and the trial's endpoints to gain better understanding of the potential connection between perceived aspects of program delivery (including how acceptable, appropriate and feasible different participant groups felt HARPdoc intervention to be) and the biomedical outcomes, including any anticipated reduction in SH in either group. We intend for our hybrid approach to inform the development of an implementation blueprint that can guide future program implementation beyond the trial.

## Aim and objectives

Our aim was to assess the perceptions of acceptability, appropriateness, and feasibility (30) of HARPdoc intervention, alongside its comparator BGAT, within the reported clinical trial (18, 31). We hypothesised that:

I) HARPdoc will be perceived more acceptable, appropriate, feasible than BGAT by HARPdoc participants, relatives, and healthcare professionals.
II) Higher perceptions of acceptability, appropriateness and feasibility will be associated with reduced rate of SH events and improved cognitive, emotional, and mental health outcomes, regardless of the assignment to the intervention.
III) The effect of HARPdoc intervention (vs. BGAT) on primary and secondary clinical outcomes will be mediated via perceptions of their acceptability, appropriateness, and feasibility.

## Methods

### Design

The HARPdoc trial was an effectiveness-implementation hybrid randomized clinical trial of the HARPdoc and BGAT programs in people with type 1 diabetes and problematic hypoglycemia. In the trial (19), a total of 99 intervention participants were recruited and randomized to one of two interventions, HARPdoc or BGAT, and were followed-up for up to 24 months. Detailed study protocols for both the effectiveness and implementation parts of the trial have been published previously: Amiel et al. 2022 (18); Soukup et al. 2019 (31), respectively. Clinical effectiveness findings have been published by Amiel et al. 2022 (19) and cost effectiveness by Healey et al. 2024 (22) (trial registration number: NCT02940873).

### Setting

The study took place in the UK and USA between July 2018 and December 2019. It included four diabetes centers across four National Health Service (NHS) Trusts in England, UK – two in the greater London area which together formed one trial center, one in Dorset, and one in South Yorkshire. In the USA, the trial included a single diabetes center in Massachusetts.

### Participants

The research participants comprised HARPdoc and BGAT intervention participants who were adults with type 1 diabetes with problematic hypoglycemia, as well as the diabetes HCPs who were involved in the delivery and supporting of the programs across the hospital sites as part of the RCT. Family and friends of HARPdoc intervention participants were invited to take part in their treatment and were given the opportunity to complete a survey on implementation as well. We refer to the combined intervention participants, HCPs, and relatives as all participant groups.

Participants for both HARPdoc and BGAT were included if they had a diagnosis of type 1 diabetes for at least 4 years, had completed education in flexible insulin therapy, had ongoing specialist care, and used either a multiple daily insulin injection regimen or an insulin pump. Exclusion criteria included type 2 diabetes, no previous education in flexible insulin therapy, pregnancy, severe mental illness, and other co-morbidities (see Amiel et al. 2022 (19) for additional details). For the implementation part of the research, there were no additional inclusion and exclusion criteria; all trial intervention participants and HCPs were invited to take part.

Severe hypoglycaemia (SH) was defined as an episode requiring assistance from another person to actively administer carbohydrate, glucagon, or other corrective actions, and may include episodes involving loss of consciousness or seizure. Impaired awareness of hypoglycaemia (IAH) was defined using established clinical criteria, including a Gold and/or Clarke score of ≥4, as specified in the trial protocol (18, 19).

### Intervention

The HARPdoc intervention was delivered to small groups of 4–8 intervention participants in four full day sessions held over 6 weeks by two diabetes educators including nurses and dietitians trained to proficiency in HARPdoc delivery and supervised by a clinical psychologist. Two one-to-one sessions between participant and one educator were held in weeks four and five and relatives were invited to the final full day group session in week six of the program. There were group follow-ups, lasting two hours, at 3 and 6 months. The program takes a psychological approach using principles of motivational interviewing and cognitive behavioral theory and addressing cognitive barriers to hypoglycemia avoidance (32). The comparator program, BGAT, was adapted to also run over 6 weeks. The small group meetings were held as two (morning and afternoon) two-hour sessions in one day, in weeks 1, 2, 3 and 6, matching the scheduling of the HARPdoc program full-day sessions and were facilitated by one educator. See the published protocol (18) for detailed description of the clinical interventions.

### Perceived implementation outcomes

After completing their educational programs, participants in the HARPdoc and BGAT programs, their relatives and all HCPs involved in program delivery were surveyed on their perceptions of acceptability, feasibility and appropriateness of the programs using previously validated survey instruments (30). All responders (intervention participants for both HARPdoc and BGAT, HCPs, and relatives) completed the same survey. Perceived acceptability, appropriateness, and feasibility were assessed using the Acceptability of Intervention Measure (AIM), the Intervention Appropriateness Measure (IAM), and the Feasibility of Intervention Measure (FIM) surveys respectively (30). Each of these measures consists of 4 questions with responses ranging from 1 (completely disagree) to 5 (completely agree). The score for each is calculated by averaging the responses to the 4 questions. Responses were scored on a 5-point Likert scale, with higher scores reflecting stronger agreement with the construct assessed i.e., perceived acceptability, appropriateness, or feasibility (30).

### Clinical, cognitive, emotional, and mental health outcomes

The rate of SH over the previous 12 months at 12- and 24-months post randomisation was assessed using anonymised recall forms completed by participants and was the primary clinical outcome in the trial. Where data were missing from the anonymised recall form, an open recall form was used if available, with the participant's knowledge and consent. These forms were specifically developed for the study to capture participant-reported SH events over the preceding 12-month period. Cognitive, emotional, and mental health secondary outcomes that showed statistically significant differences between arms in the main trial were included in the present study (19).

Cognitions around hypoglycemia were measured by the Attitudes to Awareness (A2A) questionnaire with subscales ‘Hyperglycemia Avoidance Prioritised' (HAP), ‘Hypoglycemia Concern Minimised' (HCM), ‘Asymptomatic Hypoglycemia Normalised’ (AHN), and a total score (33). The Hyperglycaemia Avoidance Scale (HAS) (34) includes behavior and worry subscales and total scores. Diabetes distress was measured by the Problem Areas in Diabetes (PAID) survey (35), and Hospital Anxiety and depression score (HADS) assessed anxiety and depression with separate scores (36). All measures are scored and analyzed on a continuous scale, with higher scores indicating worse outcomes.

### Statistical analysis

All analysis was carried out using Stata 17. Demographic and clinical characteristics were summarized for trial participants. Perceived acceptability, feasibility and appropriateness scores were summarized within the HCPs, intervention participants and relative subgroups and, overall, by treatment group. Any variables with a skewed distribution were summarized using medians and interquartile ranges (IQRs). Normally distributed variables were summarized with means and standard deviations (SD). To assess whether the subset of trial participants who provided implementation data reflected the wider study sample, we summarized intervention participant characteristics for all trial participants. We also performed chi-square tests for equality of means and nonparametric equality-of-medians tests for intervention participants' characteristics, comparing those who provided implementation data with those who did not. All *p*-values were two-tailed, and statistical significance was set at a *p*-value less than 0.05.

To address our first hypothesis, we compared perceived acceptability, feasibility, and appropriateness outcomes between program groups for only intervention participants, only HCPs, and all participant groups combined. To account for skewness in the responses to all implementation outcomes, quantile regression models were used to estimate median differences 95% confidence intervals (CIs). Separate analyses for each outcome were modelled with perceived acceptability, appropriateness, or feasibility as the dependent variable and assigned intervention as the independent variable. Due to small sample size, no other variables were included in the model. Three versions of this model were used: one included intervention participants and HCPs in both treatment arms as well as relatives of the participants in the HARPdoc program (i.e., all participant groups), the second only included HCP, and the third only included intervention participants.

To address our second hypothesis, we modelled perceptions of acceptability, feasibility, and appropriateness of the interventions as the independent variables and rate of SH events over the previous 12 months as the dependent variable, adjusting for baseline SH event rates with the use of negative binomial regression models. We calculated incidence rate ratios (IRR) and corresponding 95% CIs separately for each implementation measure for the primary outcome at 12 and at 24 months. For all secondary clinical outcomes, we used linear regression models with each outcome as the dependent variable, intervention as the independent variable, adjusted baseline measurements for each outcome. Estimates were calculated at both 12 and 24 months for each variable. Effect sizes were then standardized by dividing estimates by baseline standard deviation and plotted for each outcome.

**Figure 1:**
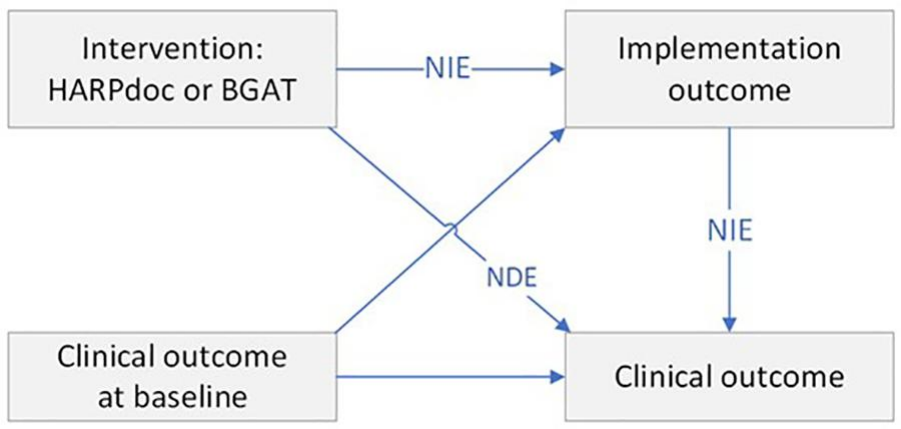
Directed acyclic graph of the model used to assess if the effect of HARPdoc intervention on clinical outcomes (attitudes to awareness, problem areas in diabetes, anxiety and depression) was mediated by implementation outcomes (acceptability, appropriateness and feasibility). All combinations of clinical and implementation outcomes were modelled seperately. NIE, natural indirect effects; NDE, natural direct effects. Unlabelled arrows show how we adjusted for baseline clinical measures when estimating both implementation and clinical outcomes.

To address our third hypothesis, we conducted a separate mediation analysis for each secondary clinical outcome that was statistically significant (*p* < 0.05). Outcomes considered were Attitudes to Awareness- Hyperglycaemia Avoidance Prioritised, Problem Areas in Diabetes, and both Hospital Anxiety and Depression. All outcomes are considered only at 12 months. Each model included one of the outcomes listed above; assigned intervention as the independent variable; AIM, FIM, or IAM as a mediator; and the relevant outcome at baseline as an independent variable. A parametric regression mediation approach (paramed package in [StataCorp]) was used to estimate the total effect, the natural indirect effects (NIE), and natural direct effects (NDE). Linear regression models were fitted for both the mediator and outcome in each analysis - see the directed acyclic graph in Figure 1. The NDE represented the effect of HARPdoc program on the primary and secondary outcomes after mediated effects via the AIM, FIM, or IAM scores were taken into account. The NIE represented the effect of the HARPdoc program on an outcome via a mediator. To further quantify the magnitude of mediation, the study estimated the proportion of the effect mediated by IAM, FIM, and AIM (NIE/[NDE + NIE]). Bootstrapping using 500 replications was used to estimate the appropriate SEs for the indirect effects, and also used for the direct effects.

## Results

### Recruitment and response rates

As part of the RCT, there were 99 intervention participants randomized, and the full sample size calculation and randomization details can be found in Amiel at al (19). All 99 study participants were approached and asked to fill out the implementation survey (30); of those, a subset of 45 (45.5%) responded and completed the survey within 3 months of post-program completion.

We also approached all 27 HCPs involved in the delivery and supporting of the HARPdoc and BGAT programs with the implementation survey, and all HPCs responded and completed it (response rate 100%). A sample of 6 relatives of HARPdoc educated participants also completed implementation surveys.

### Participant characteristics

**Figure 2:**
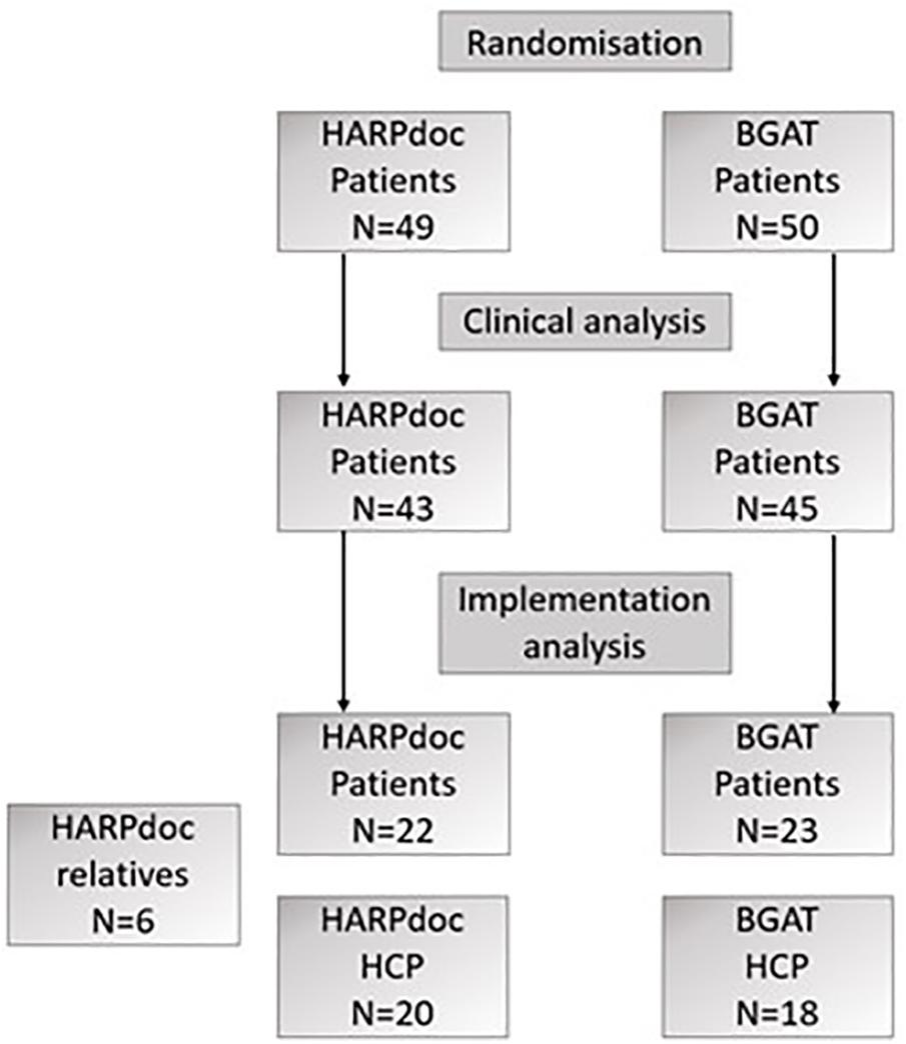
Simplified flowchart showing how intervention participants were included in the analyses. See Supplementary Appendix B for full consort diagram.

Of the 49 RCT participants assigned to HARPdoc intervention, 22 (45%) provided implementation outcome data within 3 months of post program delivery; 23 of the 50 intervention participants (46%) assigned to BGAT provided implementation outcome data. The flow of participants through the trial is shown in Figure 2, and a complete Consort diagram can be found in Supplementary Appendix B.

Table 1 provides information about baseline characteristics by treatment group for all RCT participants and for those included in the implementation analyses. Within the implementation subset the mean (±SD) age was 57.1 ± 12.6 years, 52% were female, 92.5% were Caucasian, and 72.5% were from the UK, with remaining participants from the USA. The mean ± SD duration of type 1 diabetes was 38.0 ± 16.1 years, with 62.5% of intervention participants experiencing SH for 10 or more years. In the table presented in Supplementary Appendix C, we compared the sample of intervention participants who provided implementation data to RCT participants who did not provide implementation data. Those who provided implementation data were less likely to be from the US (10.2% vs. 27.5%, *p* = 0.025), and had an older median age of 59.5 compared to 54.0 (*p* = 0.033). Aside from these differences, we found that all other baseline characteristics were similar.

**Table 1:** Demographic characteristics of HARPdoc trial participants with clinical (RCT) data only and participants with both clinical and implementation (iS) data.

Measure	HARPdoc IS	HARPdoc RCT	BGAT IS	BGAT RCT	Total IS	Total RCT
N	19	49	21	50	45	99
Age, mean (sd)	61.9 (8.9)	56.3 (12.2)	52.7 (14.0)	51.4 (14.1)	57.1 (12.6)	53.8 (13.4)
Sex, *n* (%) female	11 (57.9)	29 (59.2)	10 (47.6)	26 (52.0)	21 (52.5)	55 (55.6)
Ethnicity, *n* (%)						
Caucasian	17 (89.5)	46 (93.9)	20 (95.2)	49 (98.0)	37 (92.5)	95 (96.0)
African/Caribbean	0	0	1 (4.8)	1 (2.0)	1 (2.5)	1 (1.0)
Hispanic	1 (5.3)	1 (2.0)	0	0	1 (2.5)	1 (1.0)
Mixed	1 (5.3)	1 (2.0)	0	0	1 (2.5)	1 (1.0)
Other	0	1 (2.0)	0	0	0	1 (1.0)
BMI, mean (sd)	27.2 (4.1)	27.0 (5.5)	25.1 (4.3)	25.8 (4.4)	26.1 (4.3)	26.4 (4.9)
Duration of type 1 diabetes (years), mean (sd)	39.4 (16.6)	38.1 (16.0)	36.7 (16.0)	33.6 (14.7)	38.0 (16.1)	35.9 (15.4)
Glycated Haemoglobin % (HbA1c)	7.4 (1.3)	7.4 (1.1)	7.4 (1.2)	7.4 (1.3)	7.4 (1.2)	7.4 (1.2)
Duration of experience of severe hypoglycemia, *n* (%)						
<2 years	0	2 (4.1)	0	2 (4.0)	0	4 (4.0)
≥2 years to <5 years	5 (26.3)	14 (28.6)	4 (19.0)	8 (16.0)	9 (22.5)	22 (22.2)
≥5 years to <10 years	4 (21.1)	10 (20.4)	2 (9.5)	9 (18.0)	6 (15.0)	19 (19.2)
≥10 years	10 (52.6)	23 (46.9)	15 (71.4)	31 (62.0)	25 (62.5)	54 (54.5)
Baseline rate of SH, median (p25, p75)	4 (2, 12)	5 (2, 11)	4 (2, 10)	5 (2, 12)	4 (2, 11)	5 (2, 12)
Diabetes complications and co-morbidities						
Pancreas transplant, *n* (%)	0	2 (4.1)	0	0	0	2 (2.0)
Islet transplant, *n* (%)	0	0	0	0	0	0
Renal Transplant, *n* (%)	0	1 (2.0)	0	0	0	1 (1.0)
Symptomatic (peripheral) Neuropathy, *n* (%)	6 (31.6)	11 (22.4)	4 (19.0)	10 (20.0)	10 (25.0)	21 (21.2)
Retinopathy, *n* (%)	14 (73.7)	34 (70.8)	15 (71.4)	37 (74.0)	29 (72.5)	71 (72.4)
Technology use						
Insulin pump, *n* (%)	9 (47.4)	23 (48.9)	12 (57.1)	22 (44.0)	21 (52.5)	45 (46.4)
Pump with automated suspend feature, *n* (%)	2 (11.1)	8 (17.4)	4 (19.0)	7 (14.0)	6 (15.4)	15 (15.6)
Continuous glucose monitoring (CGM), *n* (%)	9 (47.4)	17 (36.2)	7 (35.0)	17 (34.7)	16 (41.0)	34 (35.4)
Psychiatric or psychological therapies, *n* (%)	2 (10.5)	4 (8.5)	1 (4.8)	3 (6.0)	3 (7.5)	7 (7.2)
Retrospectively intermittently (Flash) glucose monitoring, *n* (%)	1 (5.3)	3 (6.4)	2 (9.5)	9 (18.0)	3 (7.5)	12 (12.4)
Currently using Bolus Advisor, *n* (%)	12 (63.2)	27 (57.4)	8 (38.1)	16 (32.0)	20 (50.0)	43 (44.3)
Previous programs attended						
DAFNE	9 (47.4)	28 (60.9)	11 (52.4)	27 (55.1)	20 (50.0)	55 (57.9)
BERTIE	3 (15.8)	6 (13.0)	5 (23.8)	10 (20.4)	8 (20.0)	16 (16.8)
DO IT	4 (21.1)	4 (8.7)	3 (14.3)	4 (8.2)	7 (17.5)	8 (8.4)
Other structured programs	1 (5.3)	4 (8.7)	0	1 (2.0)	1 (2.5)	5 (5.3)
Other education	2 (10.5)	4 (8.7)	2 (9.5)	7 (14.3)	4 (10.0)	11 (11.6)
Stratification factors by arm						
Technology user vs MDI and HBGM, *n* (%)						
MDI and HBGM	9 (47.4)	21 (42.9)	7 (33.3)	23 (46.0)	16 (40.0)	44 (44.4)
Country, *n* (%)						
UK	13 (68.4)	41 (83.7)	16 (76.2)	41 (82.0)	29 (72.5)	82 (82.8)
USA	6 (31.6)	8 (16.3)	5 (23.8)	9 (18.0)	11 (27.5)	17 (17.2)

IS, completed implementation survey; RCT, participated in randomized controlled trial. All participants of the RCT are summarized in the RCT column and the subset who completed implementation outcome surveys (of acceptability, appropriateness, and feasibility) are summarized in the IS column.

### Descriptive statistics summarizing outcomes at all time points

Within the subset of those who provided implementation data, the intervention participants in the two treatment groups both reported a median of 4 SH episodes over the previous 12 months at baseline, with a median of 0–1 at both the 12- and 24-month follow-ups. The patient-reported clinical outcomes were similar between treatment groups. See Table 2 for further detail.

**Table 2:** Medians, 25th percentile, and 75th percentile for clinical and implementation outcomes at 0, 12, and 24 months in the implementation subset, by treatment group: BGAT or HARPdoc interventions.

Measure, median (IQR)	Baseline		12 months		24 months	
N	BGAT (*n* = 21)	HARPdoc (*n* = 19)	BGAT (*n* = 21)	HARPdoc (*n* = 19)	BGAT (*n* = 19)	HARPdoc (*n* = 19)
Severe hypoglycemia (SH) episodes in last 12 months	4.0 (2.0, 10.0)	4.0 (2.0, 12.0)	0.0 (0.0, 3.0)	1.0 (0.0, 5.0)	0.0 (0.0, 1.0)	1.0 (0.0, 2.0)
Attitudes to Awareness (A2A)						
A2A (hyperglycaemia avoidance prioritised score)	6.0 (5.0, 7.0)	6.0 (4.0, 9.0)	5.0 (3.0, 7.0)	4.0 (2.0, 8.0)	5.5 (4.0, 8.0)	4.0 (1.0, 5.0)
A2A (hypoglycemia concern minimised score)	3.0 (2.0, 4.0)	2.0 (1.0, 3.0)	2.0 (1.0, 3.0)	2.0 (1.0, 3.0)	3.0 (2.0, 5.0)	2.0 (1.0, 3.0)
A2A (asymptomatic hypoglycemia normalised)	1.0 (0.0, 3.0)	2.0 (1.0, 3.0)	1.0 (0.0, 2.0)	0.0 (0.0, 1.0)	0.0 (0.0, 1.0)	1.0 (0.0, 1.0)
A2A total	11.0 (8.0, 14.0)	10.0 (8.0, 13.7)	8.0 (6.0, 11.0)	7.0 (4.0, 10.3)	9.5 (7.0, 12.0)	6.0 (4.0, 8.0)
Hypoglycemia Fear Score (HFS-II)						
HFS-II behavior score	1.1 (0.9, 1.7)	1.2 (0.7, 1.5)	1.3 (0.6, 1.8)	0.9 (0.6, 1.3)	1.0 (0.7, 2.1)	1.0 (0.5, 1.4)
HFS-II worry score	1.4 (1.2, 2.1)	1.6 (1.2, 1.9)	1.4 (0.7, 2.0)	1.2 (0.9, 2.0)	1.1 (0.5, 1.6)	1.0 (0.3, 1.5)
HFS total score	1.2 (1.1, 1.9)	1.4 (1.0, 1.8)	1.3 (0.6, 1.8)	1.1 (0.8, 1.6)	1.0 (0.5, 1.5)	1.0 (0.5, 1.4)
Hyperglycaemia Avoidance Scale (HAS)						
HAS behavior score	1.8 (1.5, 2.1)	1.6 (1.4, 2.0)	1.6 (1.3, 2.1)	1.3 (1.0, 1.9)	1.5 (1.3, 2.0)	1.3 (1.0, 1.6)
HAS worry score	1.8 (1.6, 2.4)	2.1 (1.5, 2.6)	2.0 (1.6, 2.5)	1.8 (1.2, 2.1)	2.0 (1.7, 2.4)	1.8 (1.0, 2.0)
HAS total score	1.9 (1.4, 2.3)	1.9 (1.5, 2.1)	1.7 (1.4, 2.3)	1.6 (1.2, 2.1)	1.6 (1.3, 2.1)	1.5 (1.0, 1.8)
Diabetes distress (Problem Areas in Diabetes)	27.5 (13.8, 47.5)	26.3 (12.5, 46.3)	32.5 (18.8, 40.0)	22.5 (11.3, 43.8)	21.9 (11.3, 48.8)	13.2 (6.3, 31.3)
Hospital Anxiety and depression score (HADS)						
HADS anxiety score	8.0 (5.0, 12.0)	6.0 (4.0, 11.0)	8.0 (5.0, 11.0)	5.0 (1.0, 9.0)	6.5 (4.0, 11.0)	3.0 (1.0, 7.0)
HADS depression score	6.0 (4.0, 9.0)	3.0 (1.0, 8.0)	6.0 (4.0, 9.0)	2.0 (1.0, 5.0)	5.5 (4.0, 10.0)	3.0 (1.0, 5.0)
Acceptability (AIM)						
Intervention participants	-	-	4.38 (3.5, 5)	4 (3.5, 4)	-	-
HCP	-	-	4.63 (4, 5)	4 (3, 4.25)	-	-
Relatives	-	-	4.25 (4, 5)	-	-	-
Total	-	-	4.5 (4, 5)	4 (3.5, 4)	-	-
Appropriateness (IAM)						
Intervention participants	-	-	4.25 (3.75, 5)	3.75 (2.75, 4)	-	-
HCP	-	-	4.5 (4.13, 5)	3.75 (3, 4.25)	-	-
Relatives	-	-	4.5 (3, 5)	-	-	-
Total	-	-	4.5 (3.88, 5)	3.75 (3, 4)	-	-
Feasibility (FIM)						
Intervention participants	-	-	4.25 (3.75, 5)	4 (2.5, 4)	-	-
HCP	-	-	4 (3.75, 4.63)	3.88 (3.5, 4)	-	-
Relatives	-	-	4 (3.75, 5)	-	-	-
Total	-	-	4 (3.75, 4.75)	4 (3.25, 4)	-	-

HCP, health care professional; N, number of participants with non-missing data; IQR, interquartile range; HARPdoc, hypoglycemia awareness restoration program despite optimised self-care; BGAT, blood glucose awareness training; CI, confidence interval; AIM, acceptability of intervention measure; IAM, intervention appropriateness measure; FIM, feasibility of intervention measure.

Note that clinical outcomes are collected only for intervention participants, but implementation surveys were given to intervention participants, HCPs, and relatives.

The four items comprising each of the acceptability, appropriateness, and feasibility measures were found to be well correlated with each other and Cronbach's alpha was >0.91 for all measures. See details in Supplementary Appendix A.

#### Hypothesis 1: is HARPdoc perceived to be more acceptable, appropriate, and feasible compared to BGAT?

Results varied across the three implementation outcomes. HARPdoc had higher median scores (compared to the BGAT program) of acceptability and appropriateness subscales in at least one group, but not on feasibility.

All groups rated appropriateness higher for the HARPdoc intervention, with a median difference of 0.75 in intervention participants (95% CI 0.11, 1.39), HCP (95% CI 0.04, 1.46), and for all participant groups (95% CI 0.26, 1.24).

Intervention participants found HARPdoc intervention more acceptable with a median difference of 0.75 points (95% CI 0.33, 1.17). When all participant groups were considered, the difference was smaller at 0.50 points, but remained significant (95% CI 0.13, 0.87).

The median rating of feasibility amongst all those surveyed was 4 out of 5 points, regardless of treatment group. For only intervention participants and only HCPs, we estimated slightly higher feasibility scores in the HARPdoc group, but the differences were not significant. See comparisons for all outcomes detailed in Table 3.

**Table 3:** Comparing implementation attitudes between HARPdoc and BGAT programs for acceptability, appropriateness, and feasibility.

Implementation outcome	Group	HARPdoc N	HARPdoc median (IQR)	BGAT N	BGAT median (IQR)	Estimated median difference (95% CI)	*p*-value
AIM (acceptability)
	All participant groups	48	4.50 (4.00, 5.00)	41	4.00 (3.50, 4.00)	0.50 (0.13, 0.87)	0.009^*^
	HCP	20	4.63 (4.00, 5.00)	18	4.00 (3.00, 4.25)	0.50 (−0.27, 1.27)	0.198
	Intervention participants	19	4.75 (4.00, 5.00)	21	4.00 (3.50, 4.00)	0.75 (0.33, 1.17)	0.001^*^
IAM (appropriateness)
	All participant groups	48	4.50 (3.88, 5.00)	41	3.75 (3.00, 4.00)	0.75 (0.26, 1.24)	0.003^*^
	HCP	20	4.50 (4.13, 5.00)	18	3.75 (3.00, 4.25)	0.75 (0.04, 1.46)	0.038^*^
	Intervention participants	19	4.50 (4.00, 5.00)	21	3.75 (2.75, 4.00)	0.75 (0.11, 1.39)	0.022^*^
FIM (feasibility)
	All participant groups	48	4.00 (3.75, 4.75)	41	4.00 (3.25, 4.00)	0.00 (−0.31, 0.31)	>0.99
	HCP	20	4.00 (3.75, 4.63)	18	3.88 (3.50, 4.00)	0.25 (−0.19, 0.69)	0.251
	Intervention participants	19	4.25 (4.00, 5.00)	21	3.75 (2.50, 4.00)	0.50 (−0.21, 1.21)	0.163

AIM, acceptability of intervention measure; IAM, intervention appropriateness measure; FIM, feasibility of intervention measure; HCP, health care professional; N, number of participants with non-missing data; IQR, interquartile range; HARPdoc, hypoglycemia awareness restoration program despite optimised self-care; BGAT, blood glucose awareness training; CI, confidence interval. Perceived acceptability, appropriateness and feasibility outcomes were compared between intervention participants, health care professional groups, and all participant groups, which includes both intervention participants and HCP in both interventions as well as relatives in the HARPdoc treatment arm. Scores for each category are on a 5-point scale, with 5 indicating more favourable attitudes and 1 indicating less favourable attitudes. Differences were estimated using quantile regression.

* Statistically significant at α = 0.05.

#### Hypothesis 2: were higher perceptions of acceptability, appropriateness and feasibility associated with reduced rate of SH events and improved cognitive, emotional, and mental health outcomes, regardless of the assignment to the intervention?

We explored association between perceived acceptability, appropriateness, and feasibility and all patient-reported clinical outcomes at both 12 and 24 months. For all outcomes, lower scores indicate improvement in that area.

There was an observed decrease in the rate of SH events of 0.60, 0.67, and 0.71 at 12 months as acceptability, appropriateness, and feasibility scores increased.

At 12 months follow-up, perceived appropriateness scores were related to improved attitudes to awareness (A2A), particularly for Hypoglycemia Concern Minimised (−0.60, 95% CI −1.11, −0.09), Asymptomatic Hypoglycemia Normalised (−0.60, 95% CI −1.15, −0.04), and the total A2A score (−1.74, 95% CI −3.23, −0.25). We also observed that lower PAID and anxiety scores were linked to higher perceived appropriateness of intervention. Perceived feasibility was also linked to improved A2A total scores (−1.57, 95% CI −3.07, −0.07), PAID score (−5.25, 95% CI −9.09, −1.41), and anxiety (−1.07, 95% CI −2.03, −0.10).

**Figure 3:**
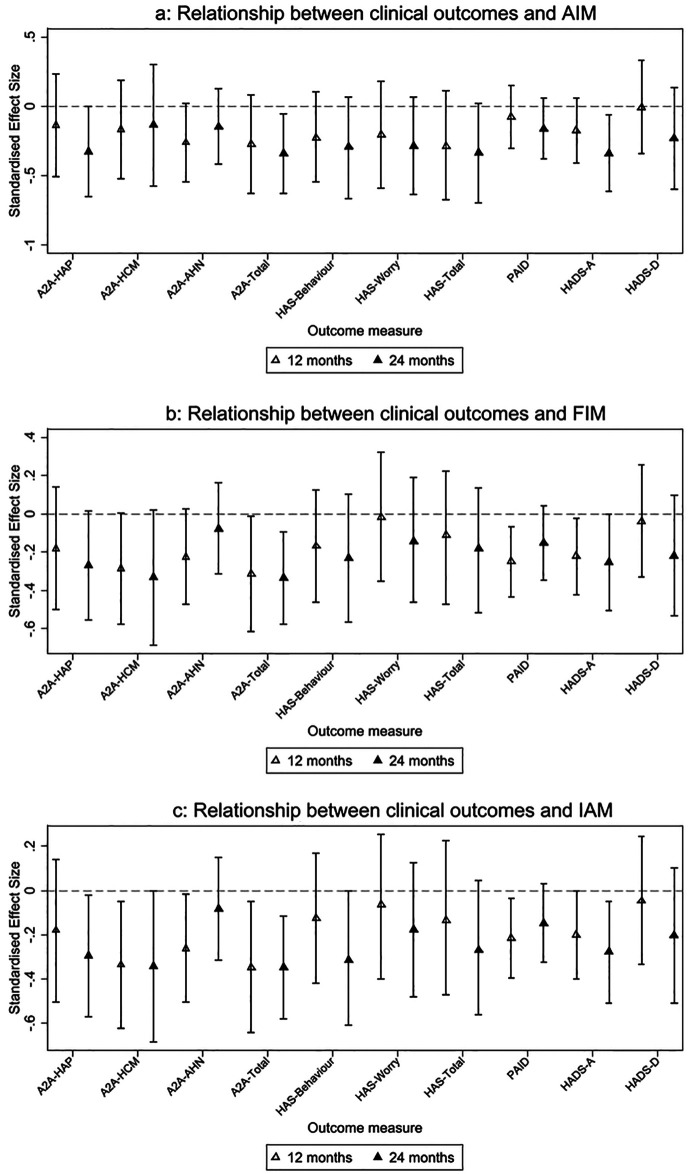
**(**a–c**)** Confidence interval plots for estimates of relationship between each implementation outcome measured and patient-reported clinical outcomes, standardised by baseline standard deviation of each clinical outcome. Each secondary mental health outcome was modelled at 12 and 24 months with AIM, FIM, and IAM scores as the independent variables in Figures 3a, b, c, respectively.

At 24 months follow-up, the A2A total scores continued to be positively linked to higher scores on the perceived acceptability, appropriateness, and feasibility scales, with a difference also being seen for acceptability at this time point. Higher scores in all three implementation outcomes were also associated with improved anxiety scores. Overall, many of the differences we saw at 12 months were sustained over 24 months. Standardized estimates as seen in confidence interval plots (Figures 3a–c) Full results can be found in Supplementary Appendix D.

#### Hypothesis 3: was the effect of HARPdoc intervention (vs. BGAT) on primary and secondary clinical outcomes mediated via perceptions of acceptability, appropriateness, and feasibility?

There was no evidence that any of the implementation outcomes mediated the effect of HARPdoc intervention on any of the clinical outcomes considered, barring PAID. There is statistical evidence of an indirect effect only of HARPdoc intervention on PAID scores when mediated by either perceived appropriateness (−5.05, 95% CI −10.56, −1.26) or perceived feasibility (−5.56, 95% CI −11.72, −1.79) ratings. There was no evidence of direct or total effects of HARPdoc intervention on PAID in the intervention participants analysed. See Supplementary Appendix E for detailed results.

## Discussion

This study represents the first randomized controlled hybrid type 2 trial that simultaneously evaluated the effectiveness and implementation of the HARPdoc intervention and its comparator BGAT.

### Hypothesis-specific findings

Consistent with our first hypothesis, both interventions were viewed positively by participants, with the HARPdoc intervention showing higher ratings of acceptability and appropriateness, with moderate differences of up to 0.75 points on a 5-point scale. While established cut-off scores for AIM, IAM, and FIM are not currently available (30), the observed difference of up to 0.75 points on a 5-point scale represents a moderate shift in perceived implementation outcomes, particularly in the context of high overall ratings (generally 3.5–4 out of 5). This corresponds to a meaningful relative change in participant perceptions, suggesting that HARPdoc was experienced as more acceptable and appropriate compared to BGAT, despite both interventions being rated positively overall. This moderate effect supports the conclusion that HARPdoc intervention was more favorably received, overall (though this finding is to be seen in the context of the BGAT intervention receiving high ratings too on the same implementation measures). Previous analysis of clinical outcomes showed that the interventions were equally effective at reducing the rate of SH (19), but if those involved in the programs find HARPdoc to be more acceptable and appropriate than BGAT, these perceptions may indicate that HARPdoc intervention would be a preferable intervention to implement. Interestingly, HARPdoc was perceived to be no less feasible than BGAT, despite the greater cost and complexity (22). Based on our analysis, we conclude that the implementation of HARPdoc intervention was generally perceived more positively than the BGAT intervention.

It is also important to consider that HARPdoc and BGAT differed in their structure and intensity, including the use of full-day sessions, structured psychological input, and one-to-one sessions in HARPdoc. These differences may have influenced participants' perceptions of acceptability and appropriateness independently of the intervention content itself. For example, increased contact time, individualised support, and the involvement of psychological expertise may enhance perceived relevance and engagement, thereby shaping implementation outcomes. As such, the observed differences in acceptability and appropriateness should be interpreted with some caution, as they may partially reflect differences in intervention intensity rather than content alone. Future research may benefit from more closely matched intervention formats or from explicitly examining the role of intervention intensity in shaping implementation perceptions.

Furthermore, consistent with our second hypothesis higher perceptions of acceptability, appropriateness and feasibility were associated with improvements in several patient-reported clinical outcomes, suggesting a potential relationship between implementation perception and clinical results. While this suggests that effective implementation may play a role in driving positive outcomes, it is also plausible that participants' perceptions were shaped by how well the interventions worked for them. As this study did not manipulate or randomise implementation strategies, we cannot determine the direction of the relationship. Future trials using implementation-focused designs could help disentangle whether positive implementation experiences lead to better outcomes, and vice versa.

Finally, we did not confirm our third hypothesis related to the question on whether the implementation perceptions mediated the effect of intervention on clinical outcomes. We did not find any evidence of mediation. We consider the answer to this objective inconclusive: our models only included a subset of the total trial participants, and the reduced size of our sample reduced the power of our estimates. This highlights the need for future research with larger sample sizes and integrated data collection across both clinical and implementation outcomes at intervention participant-level to better assess potential mediation pathways.

### Broader context and contributions

Our findings contribute to the growing body of research on implementation outcomes, aligning with the ten-year review by Proctor et al. (37), that highlights the need to explore relationships between implementation and clinical outcomes. We did not find evidence that perceived acceptability, appropriateness and feasibility scores mediated the effect of treatment on clinical outcomes, quite possibly due to our limited sample size and reduced power. We did, however, observe significant associations between higher perceived implementation scores (specifically, of acceptability, appropriateness, feasibility) and improved cognitive and mental health outcomes, supporting the potential utility of these measures as proximal indicators of success.

These findings reinforce the importance of effective implementation in enhancing outcomes and echo calls for larger, better-powered hybrid trials with integrated data collection. Future research should prioritize concurrent assessment of implementation and clinical outcomes, with the require sample size to support the multi-variable analyses of the type that we report here. By focusing on these aspects, future studies can advance understanding of how implementation processes drive clinical benefits, ultimately improving the adoption and sustainability of complex interventions like HARPdoc. This research emphasizes the dual need to evaluate and refine both the delivery and clinical outcomes of such interventions, bridging the gap between research and real-world practice.

This study is one of the first we are aware of to assess mediation pathways in an implementation-effectiveness hybrid trial, offering a methodological contribution to the field. Our results highlight the importance of effective implementation in achieving patient-centered outcomes and suggest that higher acceptability and appropriateness scores could guide the promotion and scaling of HARPdoc intervention in real-world settings. Future research should also consider longitudinal designs to examine the sustainability or maintenance of perceived acceptability, appropriateness and feasibility, and their long-term influence on clinical outcomes, addressing an important gap in the literature. These efforts can help close the implementation-effectiveness gap and provide a robust evidence base to support the broader adoption of complex interventions like HARPdoc intervention.

### Implications

Our study offers a holistic approach by considering both clinical outcomes and implementation processes, providing important insights for the development of an implementation blueprint and the expansion of HARPdoc and BGAT programs into routine healthcare services following a published clinical trial. Additionally, our findings lay the groundwork for an implementation trial, likely a hybrid type 3 (23), to assess the success of randomized implementation strategies across different hospital sites/healthcare systems, as well as the development of a clinical HARPdoc service as part of an evidence-based care pathway for people with problematic hypoglycemia in their T1D therapy. These dual implications highlight the potential of HARPdoc intervention to not only inform future research but also address an urgent clinical need for targeted interventions in this population.

This research also addresses methodological and practical gaps in evaluating and implementing complex interventions (25), emphasising the need for ongoing support and maintenance of the programs beyond the initial implementation phase. By prioritising interdisciplinary collaboration, organisational support/’buy-in', innovative research designs (e.g., hybrids) (34), standardised evaluation methods (e.g., AIM, IAM, FIM) (30), and contextual considerations, healthcare systems can enhance the successful implementation of complex interventions (23, 25, 28, 29), such as HARPdoc and BGAT programs, and improve outcomes for people with T1D and problematic hypoglycemia.

### Limitations

Our findings need to be interpreted with some limitations in mind. First, the inability to collect implementation scores from all intervention participants originally recruited into the HARPdoc trial has resulted in a reduced sample size (19).

The relatively low response rate (45.5%) may reflect methodological issues related to the timing and administration of the implementation survey. As the survey was administered separately from those measuring primary and secondary outcomes, due to distinct ethical approvals and consenting procedures, participants may not have perceived its direct relevance to the main trial. This may have reduced motivation to respond. Additionally, survey fatigue resulting from repeated data collection requests may have further contributed to lower engagement (38, 39). It is also possible that participants who completed the implementation survey differed systematically from non-responders, for example by holding more favourable perceptions of the intervention, which may introduce response bias and limit generalisability. Future hybrid trials should aim to integrate implementation and effectiveness data collection more closely to minimise burden and improve response rates. In addition, SH outcomes were based on 12-month participant recall, which may be subject to recall bias. This could lead to under- or over-estimation of SH events and should be considered when interpreting the magnitude of the observed effects.

### Future research

Future research should begin by exploring how the two programs were implemented in practice, to better understand which delivery or contextual factors may have influenced the implementation ratings observed. Future trials could also experimentally vary specific implementation strategies across study arms to examine their causal influence on implementation and clinical outcomes. Collecting implementation and effectiveness measures concurrently from all participants in a hybrid trial would then allow for better randomization and examination of causal effects in addition to observed relationships. This would provide more reliable evidence for understanding the impact of implementation. The study should also be replicated in different settings or populations to assess the generalizability of findings. This would help validate the results and provide a broader understanding of the relationships between implementation outcomes and clinical outcomes. A longitudinal study design for implementation evaluation should also be considered as it would enable follow-up over an extended period and provide a more comprehensive understanding of the effects of implementation on clinical outcomes, particularly in terms of sustainability and maintenance. Future research may consider the role of dietary management, including carbohydrate counting, as an additional component influencing outcomes in interventions targeting problematic hypoglycaemia.

## Conclusion

Our findings demonstrate that HARPdoc intervention was perceived as overall more acceptable and appropriate than BGAT, reflecting its favourable reception among participant groups. Additionally, our study documents positive associations between higher scores on perceived acceptability, appropriateness, and feasibility of the intervention and patient-reported clinical outcomes, including improvements in mental health measures such as reduced anxiety and diabetes distress. These results highlight the potential of HARPdoc intervention to address both clinical and psychological needs in people with type 1 diabetes and problematic hypoglycemia and offer a direction for similar studies to undertake larger-scale analyses of implementation and clinical outcomes.

## Data Availability

The raw data supporting the conclusions of this article will be made available by the authors, without undue reservation.
