# Supplementary material for: Psychoeducation for adults with type 1 diabetes and problematic hypoglycemia: implementation analysis of a clinical trial (HARPdoc)
Source: Front Health Serv. 2026 May 19;6:1792881. doi: 10.3389/frhs.2026.1792881 (PMC13226478; doi:10.3389/frhs.2026.1792881)
Supplement: Supplementary file 1 [file Table1.docx]

# APPENDICES

## APPENDIX A: Cronbach's alpha results for implementation data, correlation matrix for all items in implementation measures, and correlation between implementation outcome totals

Cronbach’s alpha results

| Measure | Number of items in scale | Interitem covariance | Cronbach's alpha |
| --- | --- | --- | --- |
| Acceptability scale | 4 | 0.6848 | 0.9356 |
| Appropriateness scale | 4 | 0.8458 | 0.9478 |
| Feasibility scale | 4 | 0.6601 | 0.9138 |

Correlation matrix between each set of questions in the implementation questionnaire are presented below. Darker colours represent more highly correlated item pairs.

Feasability


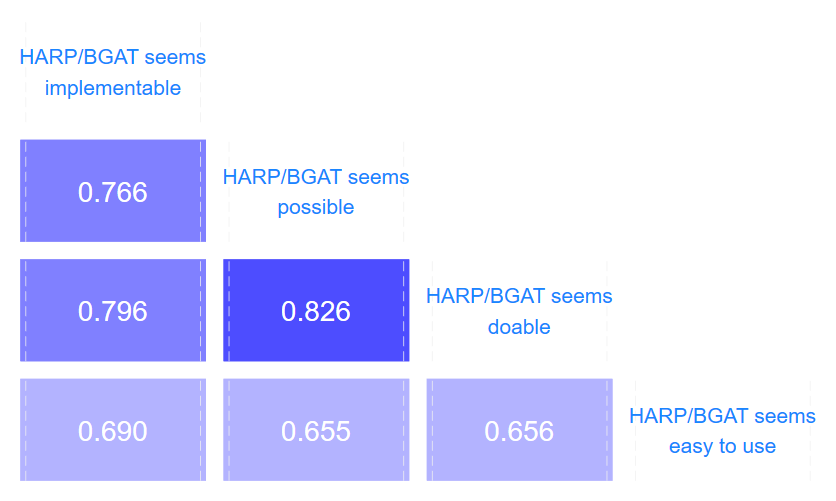


Acceptability


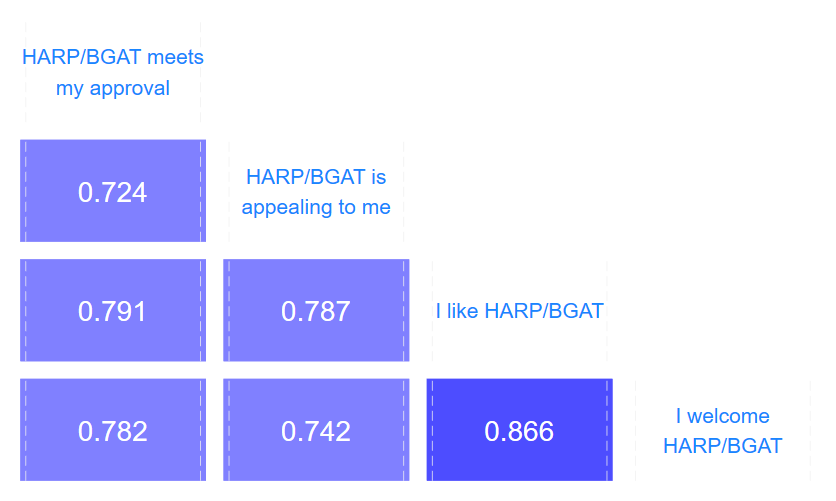


Appropriateness


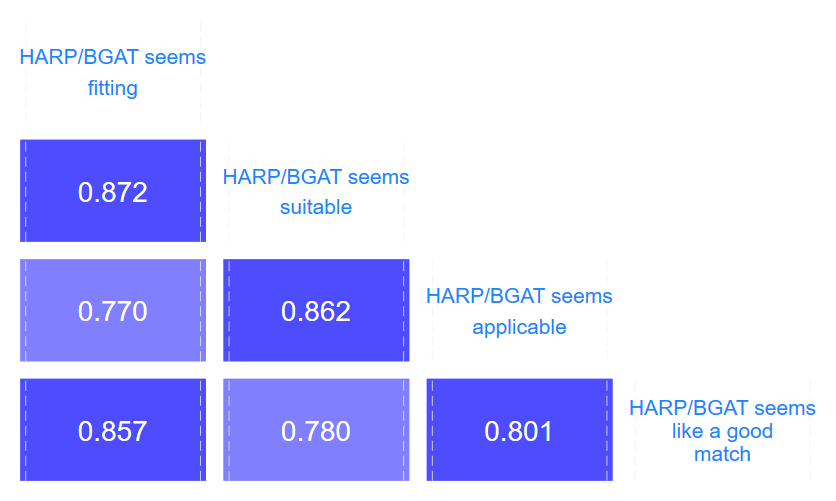


## Appendix B: Consort flow diagram

adapted from Amiel et al (20)
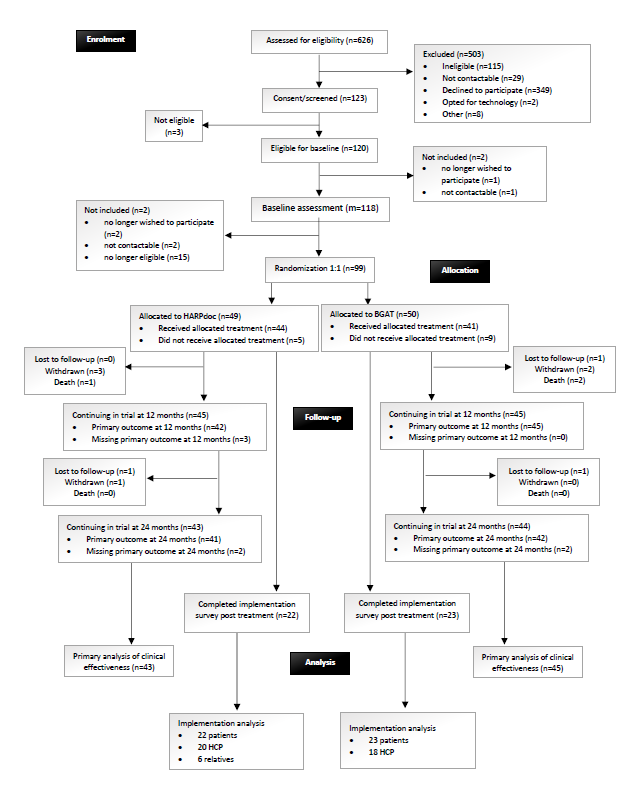


## APPENDIX C: descriptive statistics for variables of interest from participants of the HARPdoc programme who contributed implementation data compared to those who did not

|  | Clinical only | Clinical and implementation | Chi^2^ test p-value | Total |
| --- | --- | --- | --- | --- |
| Binary variables, number (%) yes | |  |  |  |
| Female | 34/59 (57.63) | 21/40 (52.50) | 0.614 | 55/99 (55.56) |
| MDI user | 28/59 (47.46) | 16/40 (40.00) | 0.464 | 44/99 (44.44) |
| Categorical variables, number (%) in each category | | |  |  |
| site |  |  |  |  |
| London | 31/59 (52.54) | 14/40 (35.00) | 0.085 | 45/99 (45.45) |
| Sheffield | 13/59 (22.03) | 7/40 (17.50) | 0.581 | 20/99 (20.20) |
| Bournemouth | 9/59 (15.25) | 8/40 (20.00) | 0.539 | 17/99 (17.17) |
| Joslin | 6/59 (10.17) | 11/40 (27.50) | 0.025 | 17/99 (17.17) |
| country |  |  |  |  |
| UK | 53/59 (89.83) | 29/40 (72.50) | 0.025 | 82/99 (82.83) |
| USA | 6/59 (10.17) | 11/40 (27.50) | 0.025 | 17/99 (17.17) |
| ethnicity |  |  |  |  |
| Caucasian | 58/59 (98.31) | 37/40 (92.50) | 0.150 | 95/99 (95.96) |
| African/Caribbean | 0/59 (0.00) | 1/40 (2.50) | 0.222 | 1/99 (1.01) |
| Hispanic | 0/59 (0.00) | 1/40 (2.50) | 0.222 | 1/99 (1.01) |
| Mixed | 0/59 (0.00) | 1/40 (2.50) | 0.222 | 1/99 (1.01) |
| Other | 1/59 (1.69) | 0/40 (0.00) | 0.408 | 1/99 (1.01) |
| Duration of hypoglycaemia | |  |  |  |
| <2 years | 4/59 (6.78) | 0/40 (0.00) | 0.093 | 4/99 (4.04) |
| 2+ years | 13/59 (22.03) | 9/40 (22.50) | 0.956 | 22/99 (22.22) |
| 5+ years | 13/59 (22.03) | 6/40 (15.00) | 0.383 | 19/99 (19.19) |
| 10+ years | 29/59 (49.15) | 25/40 (62.50) | 0.191 | 54/99 (54.55) |
| Continuous variables, median (IQR) | | | Nonparametric equality-of-medians test p-value |  |
| Age | 54.0 (42.0, 61.0) | 59.5 (48.5, 66.5) | 0.033 | 56.0 (44.0, 64.0) |
| BMI | 25.26 (22.64, 28.15) | 25.62 (22.54, 28.83) | >0.999 | 25.51 (22.63, 28.80) |
| Duration of T1D | 32.73 (22.07, 45.03) | 41.31 (22.21, 48.50) | 0.190 | 36.74 (22.07, 46.88) |
| N SH events / 12 months | 5 (2, 12) | 4 (2, 11) | 0.685 | 5 (2, 12) |
| N MH events / 4 weeks | 8 (3, 15) | 6 (3, 15) | 0.295 | 7.50 (3, 15) |
| HbA1c | 7.45 (6.50, 8.20) | 7.10 (6.50, 8.10) | 0.513 | 7.30 (6.50, 8.10) |

notes:

This table is to compare how the sample of intervention participants who provided implementation data compares to the RCT participants who did not provide implementation data.

The differences in number of intervention participants from each site between the two samples is a relevant limitation, there are not enough quantitative data in each site to compare statistically, but we did see differences between sites in the qualitative data. The difference in collection is due to inconsistencies in how and when intervention participants were given the implementation outcome surveys.

## APPENDIX D: Relationships between scores on perceived acceptability, appropriateness and feasibility and patient-reported clinical outcomes.

Implementation measures include AIM (acceptability), IAM (appropriateness), and FIM (feasibility).

Primary outcome: Results of negative binomial models exploring the relationship between implementation measures and the primary clinical outcome, adjusted for baseline SH event counts. Coefficients represent an incidence rate ratio (IRR)

All other outcomes: We used regression models to explore the relationship between implementation measures and clinical outcomes, adjusted for baseline measurements of each outcome. Coefficients represent the change in clinical outcome score per 1 point increase in implementation outcome rating. For each clinical outcome excluding the primary outcome, lower scores are healthier. Decreased scores indicate improvement in that area.

|  |  |  | 12 months |  |  | 24 months |  |
| --- | --- | --- | --- | --- | --- | --- | --- |
| Clinical outcome | Implementation measure | N | Coefficient (95% CI) | p-value | N | Coefficient (95% CI) | p-value |
| Primary Outcome  Rate of SH events over 12 months | AIM | 40 | 0.60 (0.28, 1.28) | 0.188 | 38 | 1.60 (0.79, 3.25) | 0.194 |
|  | IAM | 40 | 0.67 (0.32, 1.41) | 0.290 | 38 | 1.40 (0.69, 2.84) | 0.356 |
|  | FIM | 40 | 0.71 (0.33, 1.53) | 0.382 | 38 | 1.43 (0.70, 2.91) | 0.328 |
| A2A HAP (Attitudes to Awareness- Hyperglycaemia Avoidance Prioritised) | AIM | 40 | -0.36 (-1.33, 0.61) | 0.461 | 35 | -0.86 (-1.71, -0.01) | 0.048* |
|  | IAM | 40 | -0.47 (-1.32, 0.37) | 0.265 | 35 | -0.77 (-1.49, -0.06) | 0.035* |
|  | FIM | 40 | -0.47 (-1.31, 0.37) | 0.266 | 35 | -0.70 (-1.45, 0.04) | 0.063 |
| A2A HCM (Attitudes to Awareness- Hypoglycaemia Concern Minimised) | AIM | 40 | -0.30 (-0.94, 0.34) | 0.346 | 35 | -0.24 (-1.03, 0.54) | 0.535 |
|  | IAM | 40 | -0.60 (-1.11, -0.09) | 0.022* | 35 | -0.61 (-1.22, -0.001) | 0.049* |
|  | FIM | 40 | -0.51 (-1.03, 0.01) | 0.053 | 35 | -0.59 (-1.22, 0.04) | 0.067 |
| A2A AHN (Attitudes to Awareness- Asymptomatic Hypoglycaemia Normalised) | AIM | 40 | -0.60 (-1.25, 0.05) | 0.067 | 35 | -0.34 (-0.96, 0.28) | 0.269 |
|  | IAM | 40 | -0.60 (-1.15, -0.04) | 0.036* | 35 | -0.19 (-0.72, 0.34) | 0.463 |
|  | FIM | 40 | -0.51 (-1.07, 0.06) | 0.078 | 35 | -0.18 (-0.72, 0.37) | 0.514 |
| A2A Total (Attitudes to Awareness- Total score) | AIM | 40 | -1.37 (-3.14, 0.39) | 0.123 | 35 | -1.71 (-3.14, -0.27) | 0.021* |
|  | IAM | 40 | -1.74 (-3.23, -0.25) | 0.023* | 35 | -1.74 (-2.90, -0.59) | 0.004** |
|  | FIM | 40 | -1.57 (-3.07, -0.07) | 0.040* | 35 | -1.67 (-2.88, -0.46) | 0.008** |
| HAS (Hyperglycaemia Avoidance Survey) behaviour subscale | AIM | 40 | -0.11 (-0.26, 0.05) | 0.182 | 33 | -0.14 (-0.32, 0.03) | 0.108 |
|  | IAM | 40 | -0.06 (-0.20, 0.08) | 0.362 | 33 | -0.15 (-0.29, -0.0001) | 0.050* |
|  | FIM | 40 | -0.08 (-0.22, 0.06) | 0.254 | 33 | -0.11 (-0.27, 0.05) | 0.180 |
| HAS (Hyperglycaemia Avoidance Survey) worry subscale | AIM | 40 | -0.13 (-0.37, 0.11) | 0.273 | 35 | -0.18 (-0.40, 0.04) | 0.113 |
|  | IAM | 40 | -0.04 (-0.25, 0.16) | 0.679 | 35 | -0.11 (-0.30, 0.08) | 0.238 |
|  | FIM | 40 | -0.01 (-0.22, 0.20) | 0.921 | 35 | -0.09 (-0.29, 0.12) | 0.395 |
| HAS (Hyperglycaemia Avoidance Survey) total | AIM | 40 | -0.13 (-0.30, 0.05) | 0.142 | 34 | -0.15 (-0.31, 0.01) | 0.069 |
|  | IAM | 40 | -0.06 (-0.21, 0.10) | 0.455 | 34 | -0.12 (-0.25, 0.02) | 0.083 |
|  | FIM | 40 | -0.05 (-0.21, 0.10) | 0.492 | 34 | -0.08 (-0.23, 0.06) | 0.256 |
| PAID  (Problem Areas in Diabetes questionnaire) | AIM | 40 | -1.63 (-6.41, 3.16) | 0.496 | 35 | -3.46 (-8.10, 1.17) | 0.138 |
|  | IAM | 40 | -4.54 (-8.30, -0.77) | 0.020* | 35 | -3.13 (-6.89, 0.63) | 0.100 |
|  | FIM | 40 | -5.25 (-9.09, -1.41) | 0.009** | 35 | -3.20 (-7.25, 0.85) | 0.117 |
| Anxiety  (Hospital Anxiety and Depression) | AIM | 40 | -0.86 (-1.99, 0.27) | 0.133 | 35 | -1.65 (-2.98, -0.32) | 0.016* |
|  | IAM | 40 | -0.97 (-1.93, -0.02) | 0.046* | 35 | -1.34 (-2.46, -0.23) | 0.020* |
|  | FIM | 40 | -1.07 (-2.03, -0.10) | 0.031* | 35 | -1.22 (-2.43, -0.01) | 0.048* |
| Depression  (Hospital Anxiety and Depression) | AIM | 40 | -0.03 (-1.33, 1.27) | 0.958 | 35 | -0.89 (-2.31, 0.52) | 0.207 |
|  | IAM | 40 | -0.18 (-1.29, 0.94) | 0.749 | 35 | -0.78 (-1.95, 0.39) | 0.182 |
|  | FIM | 40 | -0.15 (-1.27, 0.98) | 0.791 | 35 | -0.84 (-2.05, 0.38) | 0.172 |

* Significant at α=0.05; ** significant at α=0.01

## APPENDIX E: Results of mediation analysis. Adjusted direct and indirect associations of HARPdoc intervention on A2A HAP, PAID, Anxiety, Depression via acceptability (AIM), appropriateness (IAM), feasibility (FIM), and total implementation rating.

| Implementation measure,  effect | A2A HAP | | PAID | | Anxiety | | Depression | |
| --- | --- | --- | --- | --- | --- | --- | --- | --- |
|  | Estimate | 95% CI | Estimate | 95% CI | Estimate | 95% CI | Estimate | 95% CI |
| AIM |  |  |  |  |  |  |  |  |
| controlled direct effect | -0.33 | -1.95, 1.46 | -4.64 | -9.65, 4.63 | -1.83 | -3.49, 0.13 | -2.28 | -4.37, 0.05 |
| natural indirect effect | -0.18 | -1.11, 0.51 | -10.56 | -3.85, 2.28 | -0.25 | -1.13, 0.21 | 0.49 | -0.32, 1.39 |
| total effect | -0.51 | -1.96, 1.11 | -10.96 | -10.96, 3.92 | -2.08 | -3.64, -0.50 | -1.79 | -3.81, 0.37 |
| IAM |  |  |  |  |  |  |  |  |
| controlled direct effect | -0.05 | -1.97, 2.20 | 1.31 | -4.64, 9.39 | -1.55 | -3.51, 0.34 | -2.31 | -4.56, -0.07 |
| natural indirect effect | -0.46 | -1.88, 0.51 | -5.05 | -10.56, -1.26 | -0.53 | -1.89, 0.35 | 0.52 | -0.67, 1.90 |
| total effect | -0.51 | -1.96, 1.11 | -3.74 | -10.96, 3.92 | -2.08 | -3.64, -0.50 | -1.79 | -3.81, 0.37 |
| FIM |  |  |  |  |  |  |  |  |
| controlled direct effect | -0.11 | -2.04, 1.87 | 1.82 | -4.78, 10.67 | -1.44 | -3.34, 0.52 | -2.38 | -4.57, -0.01 |
| natural indirect effect | -0.41 | -1.62, 0.43 | -5.56 | -11.72, -1.79 | -0.64 | -1.96, 0.19 | 0.58 | -0.64, 1.95 |
| total effect | -0.51 | -1.96, 1.11 | -3.74 | -10.96, 3.92 | -2.08 | -3.64, -0.50 | -1.79 | -3.81, 0.37 |
